# Supplementary material for: Natural Killer Cell Granule Protein (NKG7) promotes the development of vaccine-induced anti-fungal Th1 cells
Source: PLoS Pathog. 2026 Jul 31;22(7):e1014187. doi: 10.1371/journal.ppat.1014187 (PMC13446730; doi:10.1371/journal.ppat.1014187)
Supplement: Supplementary file 1 [file ppat.1014187.s006.pdf]

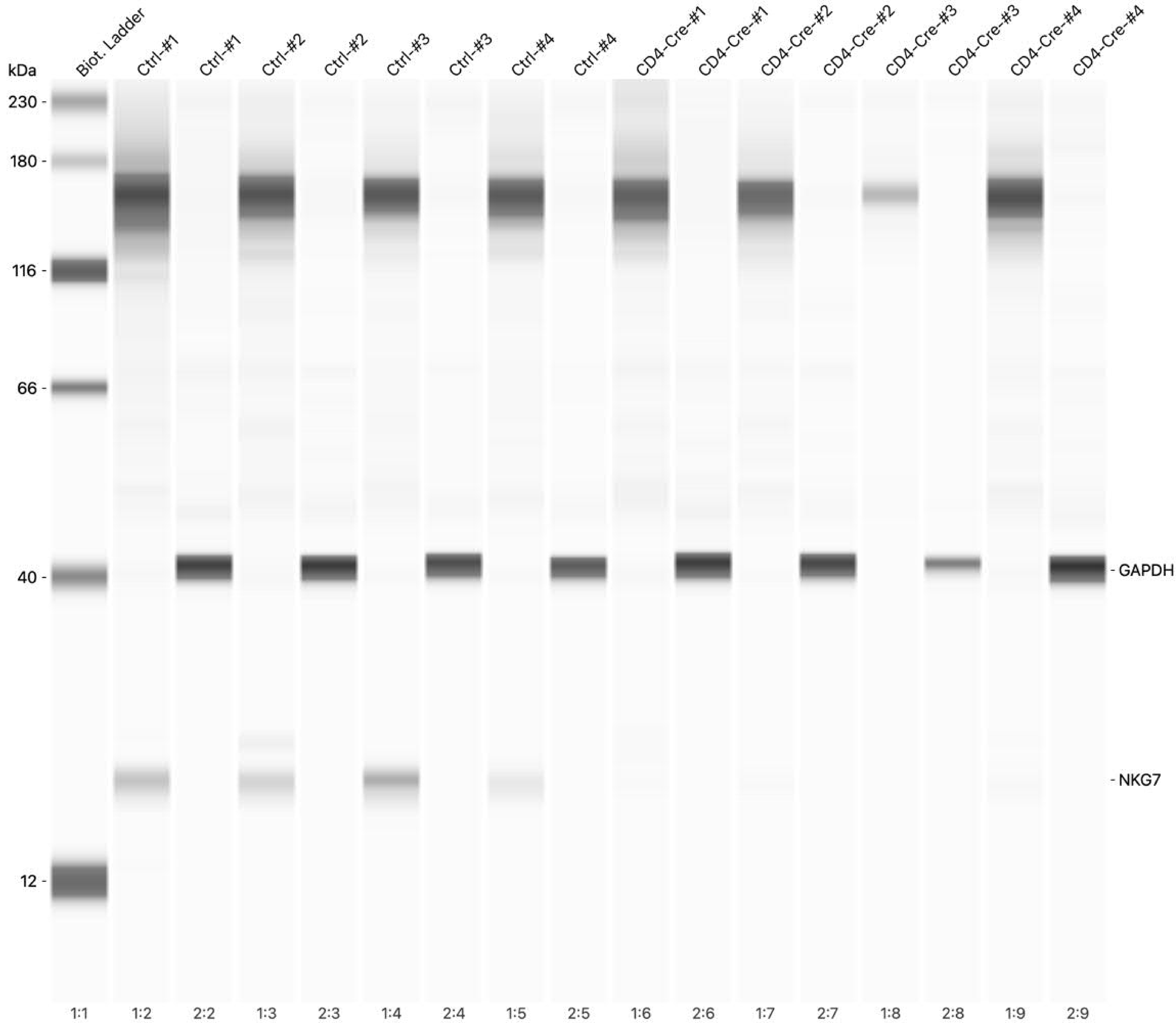
kDa
230
180
116
66
40
12
Biot. Ladder
Ctrl-#1
Ctrl-#1
Ctrl-#2
Ctrl-#2
Ctrl-#3
Ctrl-#3
Ctrl-#4
Ctrl-#4
CD4-Cre-#1
CD4-Cre-#1
CD4-Cre-#2
CD4-Cre-#2
CD4-Cre-#3
CD4-Cre-#3
CD4-Cre-#4
CD4-Cre-#4
GAPDH
NKG7
1:1
1:2
2:2
1:3
2:3
1:4
2:4
1:5
2:5
1:6
2:6
1:7
2:7
1:8
2:8
1:9
2:9
