# Supplementary material for: Natural Killer Cell Granule Protein (NKG7) promotes the development of vaccine-induced anti-fungal Th1 cells
Source: PLoS Pathog. 2026 Jul 31;22(7):e1014187. doi: 10.1371/journal.ppat.1014187 (PMC13446730; doi:10.1371/journal.ppat.1014187)
Supplement: Supplementary file 2 [file ppat.1014187.s007.docx]

**METHODS**

**Sex as a biological variable.** Both male and female C57BL/6 mice were used in vaccination experiments and similar outcomes were found.

**Fungi**

Strains included ATCC 26199 [1], a wild-type strain of *Blastomyces dermatitidis*, and the isogenic, attenuated mutant lacking BAD1, designated strain #55 [2]; *Histoplasma capsulatum* strain G217B; and *Coccidioides posadasii* isolate C735 [3] and the attenuated strain *Δcps1* [4].

**Mouse strains**

NKG7^-/-^, NKG7^flox/flox^ and NKG7-Cre mice were received from Chris Engwerda, QIMR, Berghofer Medical Research Institute, Brisbane, Australia. To generate NKG7 reporter mice we crossed NKG7-Cre with mTmG mice to yield NKG7-Cre x mTmG mice. To generate conditional knockout mice in which NKG7 is lacking in T cells, we crossed CD4-CreERT2 (Jax # 22356) with NKG7^flox/flox^ mice to yield CD4-Cre x NKG7^flox/flox^ mice. These strains of mice as well as Rag1^-/-^ (Jax #2216), Balb/C (Jax #651), 129 (Jax #2448) and C57BL6 wild type mice (Jax #664) were bred in the pathogen free Animal Breeding Core Facility at the University of Wisconsin in Madison. Male and female mice were 6-8 weeks old at the time of these experiments. Mice were lodged and cared for as per guidelines of the University of Wisconsin Animal Care Committee who approved all aspects of this work.

**Generation and purification of recombinant *Bl*-Eng2 and *Cp*-Eng2**

*Bl*-Eng2 and *Cp*-Eng2 were cloned and expressed in *P. pastoris* using standard recombinant techniques as described elsewhere [5]. Purity of recombinant Eng2 homologues were assessed by SDS-PAGE and silver staining.

**Intracellular cytokine stain.** Lung cells were harvested at day 4 to day 7 post-infection. Cells (0.5 × 10^6^ cells/ml) were stimulated for 5 hours with anti-CD3 (clone 145-2C11; 0.1μg/ml) and anti-CD28 (clone 37.51; 1μg/ml) in the presence of Golgi-Stop (BD Biosciences). Stimulation with fungal ligands yielded comparable cytokine production by transgenic T-cells compared to CD3/CD28 stimulation (data not shown). After cells were washed and stained for surface CD4 and CD8 using anti-CD4 BV395, anti-CD8 PeCy7, and anti-CD44-FITC mAbs (Pharmingen), they were fixed and permeabilized in Cytofix/Cytoperm at 4 ̊ C overnight. Permeabilized cells were stained with anti-IL-17A PE and anti-IFNγ-Alexa 700 (clone XMG1.2) conjugated mAbs (Pharmingen) in FACS buffer for 30 min at 4 ̊ C, washed, and analyzed by FACS. Cells were gated on CD4 and cytokine expression in each gate analyzed. The number of cytokine positive CD4^+^ T cells per lung was calculated by multiplying the percent of cytokine- producing cells by the number of CD4^+^ T cells in the lung.

**CRISPR/Cas9-mediated Gene Knockout**

The CRISPR/Cas9-mediated gene knockout was performed as previously described [6] with minor modifications. Three TrueGuide Synthetic single-guide RNAs (sgRNA) targeting NKG7 (CRISPR217128_SGM, CRISPR217133_SGM and CRISPR217160_SGM; Thermo Fisher Scientific) were combined, each at 150 pmol (totaling 450 pmol). The combined sgRNAs targeting NKG7 or a non-targeting control synthetic sgRNA (A35526; Thermo Fisher Scientific) were mixed with 180 pmol of TrueCut Cas9 Protein v2 (A36499; Thermo Fisher Scientific) and incubated at room temperature for 10 min to form a ribonucleoprotein (RNP) complex. A total of 10 million T cells were resuspended in 20 μL of P3 Primary Cell Nucleofector Solution (V4XP-4032; Lonza). The T cells were then mixed with the RNP complex and incubated at room temperature for 2 min. Cells were electroporated using the DS-137 program on the 4D Nucleofector system (Lonza). The probability that an allele is not cleaved by any sgRNA: Probability of not being cleaved by sgRNA1:  1 - 0.28 = 0.72. Probability of not being cleaved by sgRNA2:  1 - 0.26 = 0.74. Probability of not being cleaved by sgRNA3:  1 - 0.31 = 0.69. Combined probability of an allele not being cleaved by any sgRNA: 0.72*0.74*0.69 = 0.367. Therefore, the combined cleavage efficiency is 1-0.367=63.3%. Note that the Genomic Cleavage Detection assay often underestimates the actual rate of indel formation. Factors that account for underestimation include self-hybridization of alleles without mismatches, incomplete denaturation and reannealing during the assay, and inefficient cleavage of single nucleotide indels. Thus, the actual KO efficiency should be higher, potentially exceeding 80% or even 90%.

**Simple Western analysis**

For Simple Western analysis, cells were lysed using the Minute Total Protein Extraction Kit (Invent Biotechnologies, SD-001). Proteins were separated by size (12–230 kDa) and detected on a Jess system (ProteinSimple) using primary antibodies against NKG7 (Cell Signaling Technology, 51968; 1:10 dilution) or glyceraldehyde-3-phosphate dehydrogenase (GAPDH; Abcam, ab9485; 1:50 dilution). Protein signals were visualized and quantified using Compass software v7.0.0 (ProteinSimple).

**Vaccination and fungal infection**. Ten micrograms of *Bl*-Eng2 or 20ug *Cp*-Eng2 were loaded into GCPs and adjusted to a final volume of 200 μl with phosphate-buffered saline (PBS). Monophosporyl Lipid A (GLA) was purchased from Avanti Polar Lipids, LLC and resuspended in DMSO at 3mg/ml. GLA was mixed with GCP-*Bl*-Eng2 at 5 μg/mouse. Mice were vaccinated subcutaneously (SC) with Eng2 full length proteins three times, two weeks apart as described [7] with antigen loaded into glucan chitin particles (GCPs); controls in these experiments were GCPs loaded with mouse serum albumin (MSA) (Dr. Gary Ostroff, University of Massachusetts Chan Medical School). Two weeks after the vaccine boost, mice were challenged intratracheally (i.t.) with 2×10^4^ wild type *Bd* yeast or 100-150 *Cp* spores in 30μl PBS. At day 4-7 post-infection, lung T cell responses were analyzed and lung CFU counted. Two weeks post infection, when control or unvaccinated mice were moribund, fungal burden was determined by plating lung CFU.

**Survival**

Infected mice were sacrificed when they appeared moribund and lost more than 20% of weight.

**Statistics**

Statistical analysis was performed in Prism (GraphPad). A one-way ANOVA with Tukey’s test was used for multiple comparisons or an unpaired two-tailed t test was used for comparison between two groups, respectively. For comparison of cell count and CFU, the data were log transformed before the statistical test. Survival curves were analyzed by Mantel-Cox test. p≤ 0.05 was considered statistically significant.

**Ethics statement**

Animal studies adhered to protocol M005891 approved by the IACUC of UW-Madison. Animal studies were compliant with provisions established by the Animal Welfare Act and the Public Health Services (PHS) Policy on the Humane Care and Use of Laboratory Animals.

**REFERENCES**

1. Harvey RP, Schmid ES, Carrington CC, Stevens DA (1978) Mouse model of pulmonary blastomycosis: utility, simplicity, and quantitative parameters. American Review of Respiratory Disease 117: 695-703.

2. Brandhorst TT, Wüthrich M, Warner T, Klein B (1999) Targeted gene disruption reveals an adhesin indispensable for pathogenicity of Blastomyces dermatitidis. J Exp Med 189: 1207-1216.

3. Wüthrich M, Hung CY, Gern BH, Pick-Jacobs JC, Galles KJ, et al. (2011) A TCR Transgenic Mouse Reactive with Multiple Systemic Dimorphic Fungi. J Immunol 187: 1421-1431.

4. Shubitz LF, Powell DA, Trinh HT, Lewis ML, Orbach MJ, et al. (2018) Viable spores of Coccidioides posadasii Deltacps1 are required for vaccination and provide long lasting immunity. Vaccine 36: 3375-3380.

5. Wang H, Lee TJ, Fites SJ, Merkhofer R, Zarnowski R, et al. (2017) Ligation of Dectin-2 with a novel microbial ligand promotes adjuvant activity for vaccination. PLoS Pathog 13: e1006568.

6. Seki A, Rutz S (2018) Optimized RNP transfection for highly efficient CRISPR/Cas9-mediated gene knockout in primary T cells. J Exp Med 215: 985-997.

7. Wüthrich M, Filutowicz HI, Klein BS (2000) Mutation of the WI-1 gene yields an attenuated Blastomyces dermatitidis strain that induces host resistance. J Clin Invest 106: 1381-1389.
