# Supplementary material for: Natural Killer Cell Granule Protein (NKG7) promotes the development of vaccine-induced anti-fungal Th1 cells
Source: PLoS Pathog. 2026 Jul 31;22(7):e1014187. doi: 10.1371/journal.ppat.1014187 (PMC13446730; doi:10.1371/journal.ppat.1014187)
Supplement: Supplementary file 3 [file ppat.1014187.s008.docx]

June 5, 2026

Editors

*PlosPathogens*

**RE: Revision of manuscript PPATHOGENS-D-26-00997**
Natural Killer Cell Granule Protein (NKG7) promotes the development of vaccine-induced anti-fungal Th1 cells

Dear Editor:

We were pleased with the favorable disposition and appreciate the chance to revise our paper. We have generated new data that we include in the revised the paper in response to the reviewers’ and your requests. We provide a point-by-point response to the concerns raised. In the revision, we highlight in yellow the text with new data or discussion requested. We also provide below a bulleted summary of new results requested by reviewers that now appear either in the revision or the rebuttal letter. Due to the limited space allowable for the short report format (1800 word limit), we were not able show some of the requested but negative data in the manuscript, or to make repeated referencein to them in the results section but we now furnish them in the rebuttal letter below for the reviewers’ inspection.

- Requested lung CFU for condidional CD4-Cre x NKG7 fl/fl mice at day 6 post-infection when the cellular analysis was conducted (now shown in **Fig. 3E**).
- Requested summary of the cellular analysis illustrated in new histograms (now shown in **Fig. 4C**)
- Requested gating strategies (now shown in new supplementary **Fig. 3**)
- Requested uncropped western blot submitted as supplementary information (S1 raw image for **Fig. 2C)**.

**Part I - Summary**
Please use this section to discuss strengths/weaknesses of study, novelty/significance, general execution and scholarship.

Reviewer #1: The findings presented are novel, supported by the data presented and will open new directions for further investigation.

Reviewer #2: The introduced data are convening, the work is diligently and cleverly carried out, and the introduced work is impactful. I have very few editorial corrections and suggestions.

Reviewer #3: Open questions remain how NKG7 modulates cytokine production mechanistically (e.g. via mTORC1?) and whether NKG7 also affects the CD4 T cell longeivity as it has been proposed in case of CD8 T cells. While the authors may not solve those question in the frame of this short report, there are a few issues that need be addressed

Part II – Major Issues: Key Experiments Required for Acceptance

**Reviewer #1:** (No Response)

**Reviewer #2:** None

**Reviewer #3:**

**Query 1**. The framing of the study is very generalized. Statements such as ‘the role of Th1 cells in antifungal resistance’ should be formulated more carefully as different Th subsets protect against different types of fungal infections.

**Response:** We modified the statement in question in the first sentence of the introduction as requested.

**Query 2.** Figure 2C-F: why going through the hazzle of generating NKG7-deficient CD4 T cells by CRISPR editing, rather than using CD4 T cells from NKG7 knockout mice?

**Response:** The reviewer is correct. We could have adoptively transferred naïve CD4+ T cells from NKG7 knockout mice instead of CRISPR editing endogenous CD4+ T cells. However, we generated the data in Fig. 2C-F well *before* we received NKG7 knockout mice from our collaborator Dr. Engwerda in Australia. It took us a long time to rederive and breed the NKG7 knockout mice. We just recently accumulated experimental mice to generate the data in Fig. 2A and 2B. Importantly, the orthogonal approaches compliment one another and solidify the findings.

**Query 3.** Please provide more details and more accuracy in the results section and in the legends regarding the experimental design, mouse lines used, etc. To provide some examples: say CD4-CreERT2 throughout, not CD4-cre. Indicate how much time after vaccination mice were infected. For the latter, including schemes of the experimental schedule used in each experiment would be very helpful for the reader.

**Response:** As requested, we have changed CD4-Cre to CD4-CreERT2 throughout the manuscript. We have added schemes to each figure to indicate the schedules of vaccination, challenge and analysis. We also have added that information in the figure legends.

**Query 4.** Figure 2D-F: According to the legend, and in contrast to the methods section, animals were analyzed 7 days post-infection. By this time point, all animals in the blue and green groups have succumbed from the infection (2E). How could T cells and cfus be analyzed at this timepoint then?

**Response:** We infected two cohorts of Rag1^-/-^ mice that received CRISPR edited CD4^+^ T cells. One cohort was analyzed for intracellular cytokines and lung CFU at day 7 post-infection and a separate cohort was analyzed for survival. We have now clarified that informatin in the figure legend for Fig. 2.

**Query 5.** Figure 3D: add a summary plot for tetramer+ cells. What about fungal loads?

**Response:** Vaccinated mice with NKG7 deficient T cells had higher lung CFU than mice with NKG7-sufficient T cells. We have added these new data in Fig. 3E and mention them in the corresponding figure legend and the results. The frequency and number of tetramer positive T cells was not different between vaccinated mice with NKG7-deficient and -sufficient T cells. Thus, we chose not to show these data in the manuscript. However, we furnish the data here (see below) for the reviewer’s information.

**Query 6.** Likewise in 4C: why not show summary plots for cytokine+ populations (with individual symbols for individual animals), as done in Fig. 3?

**Response:** We performed the analysis the reviewer requested. We now show the percentages of cytokine producing T cells that were significantly different between NKG7 deficient and -sufficient T cells in Fig. 4C. The percentages of tetramer positive T cells and TNF+ and GM-CSF+ T cells from NKG7 deficient and -sufficient T cells are not included in the revised manuscript because of insignificant differences but we furnish those histograms below for the reviewer’s information.

**Query 7.** The authors claim that NKG7 regulates T cell plasticity based on a shift in IFNg- vs IL-17-producing T cells at 6 months post-vaccination. Experiments with IL-17 fate reporter mice would be required to proof that NKG7-dependent IFNg production is indeed the result of plastic ex-Th17 cells. The authors should at least tune down their conclusions. The same applies to the mentioning of memory as long as they do not show any memory marker expression by the T cells.

**Response:** As per reviewer’s suggestion, we have toned down our conclusions and point out the necessity to conduct corresponding experiments with IL-17 reporter mice outside the scope of this work (see lines 166 to 168).

**Query 8.** All flow cytometry plots: indicate on what population the displayed plots have been gated. Provide the full gating strategy in the supplementary figures, including scatter and exclusion of dead cells and doublets.

**Response:** As requested, we now indicate what population the displayed plots have been gated on in all the figures. We also have prepared a new supplementary figure (SFig.3) that displays the gating strategy including scatter and exclusion of dead cells and doublets.

**Query 9.** All figure legends: include the number of samples per group, whether mean+SD or mean+SEM are shown, and whether data are from a single experiment or pooled from n independent experiments. The statistical tests used are abbreviated (e.g., Anova-test, instead of one-way ANOVA or two-way ANOVA)

**Response:** We have added the information requested.

**Query 10.** “Tis” should be explained (e.g. in the sentence “Among16 distinct clusters of cells, NKG7 was expressed mainly in SC vaccinated mice by T cells that expressed IFN-g, e.g. NK-like Th1 cells (nTh1 cells), Tis cells, and clusters 1, 3, 4 and 5 of the Th1/Th17 cell spectrum”).

**Response:** We now spell out type1 interferon signature (Tis) cells and provide the reference where we published our previous work (Woodring et al., iScience25, 104934 September16, 2022).

Part III – Minor Issues: Editorial and Data Presentation Modifications
Please use this section for editorial suggestions as well as relatively minor modifications of existing data that would enhance clarity.

Reviewer #1:

**Query 1.** Figure legends for figures 2-4 are lacking details on how many mice were used per group, how many times were the experiments performed. Details on what statistical analysis was applied to each panel are not fully described for all data sets presented. Please revise and add this important information.

**Response:** We have added the information requested.

**Query 2.** Figure 2A shows a line between Vaccinated NKG7+ mice and vaccinated NKG7- mice together with an asterisk to denote statistical significance. Were the other groups compared for statistically significant differences? The naive NKG7- groups appears to have about a log less fungal burden that the WT naïve control. Please clarify in the legend.

**Response:** We now indicate the differences in lung CFU between all the groups that were significant. Specifically, the lung CFU between unvaccinated wild type mice and NKG7-deficient mice were significant at p=0.04. We indicate the statistical difference in the revised Fig. 2A.

**Query 3**. The authors mentioned that targeting NKG7 did not affect frequency of tetramer+ cells after vaccination. Did they quantitate total numbers of antigen-specific cells?

**Response:** Yes, we calculated the number of tetramer+ T cells in Fig. 3. They were not significantly different between vaccinated mice with NKG7-deficient T cells compared to NKG7-sufficient T cells. Thus, we chose not to include them in the manuscript. However, we furnish these data below for the reviewer’s information.

**Query 4.** For the studies with adoptive transfer of CRISP edited NKG7+ or NKG7- into RAG hosts, did the authors examine whether the frequency and total number of vaccine-elicited T cells was comparable regardless of NKG7 expression?

**Response:** Yes, we did perform the analysis requested. In the figure below, we show that the frequencies and numbers of adoptively transferred NGK7-deficient and -sufficient T cells were similar in the lungs of the RAG1^-/-^ recipient mice at day 7 postinfection. Because of the limited space available in the format of the “Short Report”, we chose not to show these data. We also feel that these data would distract from the main point of this experiment: the impediment of IFNγ^+^ T cells due to the T cell intrinsic absence of NKG7.

**Query 5.** Do the authors have a hypothesis on how NKG7 is acting to shape IFN-g expression? Several studies on CD8 T cells and in a model of Leishmania are cited but I was left wondering about how is NKG7 mediating the effects reported in the current study. I understand that experimentation to address this is beyond the scope of the current study but it would be helpful to include some thoughts about how it could be acting.

**Response:** We hypothesize that NKG7 might be required for transcription or exocytosis of IFN-γ. We also consider that NKG7 might be necessary for long term persistence of memory T cells. We have added a corresponding sentence in the Results & Discussion section in lines 174 – 175.

**Reviewer #2:**

**Query 1.** How is the gene deletion reported in the text at 90% while it is reported in the figure 2 legend that the actual loss of the gene likely exceeds 80% of the transcript?

**Response:** We thank the reviewer for catching this inconsistency, which we have now corrected. The discrepancy arose because we reported an *inferred* editing efficiency; neither was a direct measurement.

To clarify what was actually measured: the genomic cleavage efficiencies of the three individual sgRNAs (28%, 26%, and 31%) are direct measurements from the Genomic Cleavage Detection (GCD) assay we performed. The combined value of 63.3% is a *calculated estimate* — the probability that an allele is cleaved by at least one of the three guides, computed from the individual efficiencies under an assumption of independent cutting. The previously stated ">90%" (text) and ">80%" (legend) were inferences based on the known tendency of the GCD assay to underestimate true indel formation; they were not measured, and we have now removed those numbers.

We did not measure functional knockout (loss of NKG7 protein or transcript) in these CRISPR-edited cells, and we therefore no longer assign a specific knockout percentage. We note that the GCD assay underestimates true indel frequency (e.g., owing to homoduplex reformation between identically edited alleles, incomplete denaturation/reannealing, and inefficient cleavage of single-nucleotide indels), and that our probabilistic calculation does not account for deletions of the region flanked by two simultaneous cut sites; for both reasons the actual editing efficiency is likely considerably higher than the calculated estimate of at least 63.3%. We used three combined guides specifically because combining multiple guides per target is known to increase the likelihood of loss-of-function editing relative to single guides in primary T cells (Seki and Rutz, 2018, our ref 12).

Importantly, our conclusions do not depend on the precise editing efficiency in this experiment. The editing was functionally effective: NKG7 editing reduced the frequency of IFN-γ⁺ cells by 70% (Fig. 2C). Moreover, the central finding is independently reproduced in an orthogonal, genetically defined system — conditional CD4-Cre × NKG7^fl/fl mice in which loss of NKG7 protein was directly confirmed by quantitative western blot (Fig. 3A). The same defect in Th1 differentiation and antifungal resistance was observed in these mice (Fig. 3C–E). The concordance of these functional and orthogonal results, not the editing-efficiency value, is therefore the basis for our conclusion.

**Query 2.** Please spellout GCP-MSA. Specifically, what is MSA?

**Response:** MSA stands for mouse serum albumin, which was used as a control protein for the loading of glucan chitin particles (GCP). We now spelled it out the first time it was mentioned in the text (in the legend of Fig2).

**Query 3.** Are the data presented in Fig2A represents an infection with B. dematitidis, while Fig2B represents an infection with C. posadasii?

**Response:** Yes, the reviewer is correct. To clarify this information for the reader, we have now labeled the two graphs accordingly in Fig. 2A and 2B.

**Query 4**. In Figure 2F, please specify what day post infection the lung CFU were processed. The same thing applies to SFig. 1A

**Response:** Lung CFU were harvested at day 7 (Fig. 2F) and day 14 post-infection (SFig. 1A). We have now added this information to the corresponding figure legends.

**Query 5.** There is no mention of Fig. 3B in the text.

**Response:** The results shown in Fig. 3B are referred to in line 135 of the combined Results & Discussion section.

**Reviewer #3:** (No Response)

We hope that the Editors agree that the paper is stronger and is now suitable for publication.

Sincerely yours,

Marcel Wuethrich, PhD
