# Supplementary material for: Natural Killer Cell Granule Protein (NKG7) promotes the development of vaccine-induced anti-fungal Th1 cells
Source: PLoS Pathog. 2026 Jul 31;22(7):e1014187. doi: 10.1371/journal.ppat.1014187 (PMC13446730; doi:10.1371/journal.ppat.1014187)
Supplement: Supplementary file 4 [file ppat.1014187.s009.docx]

July 14, 2026

Editors

*PlosPathogens*

**RE: Revision of manuscript PPATHOGENS-D-26-00997**
Natural Killer Cell Granule Protein (NKG7) promotes the development of vaccine-induced anti-fungal Th1 cells

Dear Editor:

We were pleased with the favorable disposition after the first revision and appreciate the chance to revise our paper again. We have revised the paper in response to the reviewers’ and your requests. We provide a point-by-point response to the concerns raised. In the revision, we highlight in yellow the text with new data requested. We also provide below a bulleted summary of new results requested by reviewers that now appear in the revision.

- Requested numbers of CD4+CD44+ T cells for Fig. 2D in new histogram (now shown in SFig.4)
- Requested numbers of tetramer+ T cells for Fig. 3B in new histogram (now shown in SFig. 4B)
- Requested numbers of tetramer+ T cells for Fig. 4A in new histogram (now shown in SFig. 4C)

**Part I - Summary**
Please use this section to discuss strengths/weaknesses of study, novelty/significance, general execution and scholarship.

Reviewer #1: The authors have properly addressed my previous questions

Reviewer #2: The authors addressed my concerns. I have no further questions and I recommend acceptance.

Reviewer #3: The authors have addressed all my concerns and carefully replied to my comments. I have a few remaining remarks

Part II – Major Issues: Key Experiments Required for Acceptance

**Reviewer #1:** (No Response)

**Reviewer #2:** see above

**Reviewer #3:** (No Response)

Part III – Minor Issues: Editorial and Data Presentation Modifications
Please use this section for editorial suggestions as well as relatively minor modifications of existing data that would enhance clarity.

**Reviewer #1:** (No Response)

**Reviewer #2:** (No Response)

**Reviewer #3:**

Query 1: Rgd my original comment #3: In Fig. 3 and Fig. 4, panels are still labelled with CD4 Cre, instead of CD4-CreERT2

Response: We furnished that information in the initial revision. To retain clarity and simplicity of both figures, we prefer not to “clutter” the figures with the requested information but we provide it highlighted in the legends of the corresponding figures.

Query 2: Rgd my original comment #4: I understand that the authors used a separate cohort of mice for the survival experiment. But the question remains: if the data shown in Figure 2E are representative and reproducible, mice in the non-vaccinated group in and in the vaccinated -NKG7 group all succumb within 6 days. How could the analyses shown in Figure 2D and 2F be conducted on day 7, i.e. a time point when animals from two out of three groups were no longer alive.

Please also adjust the methods section regarding the time point of analysis (it currently states “Lung cells were harvested at day 4 post-infection”, which seems not to be correct ) and describe how the endpoint was defined in survival experiments (e.g. spontaneous death or culling of the animals when reaching a certain loss of weight)

Response: The survival and lung CFU experiments were conducted with separate cohorts of mice on separate days using separate preparations of live *B. dermatitidis* yeast. This resulted in a slightly different time frame when control mice became moribund.

We modified the Methods section to reflect the timing of the harvest of lung cells on page 4, second line from the bottom of the methods section. We now also describe in the Methods how the endpoint for the survival outcomes was defined.

Query 3: Rgd my original comment #5: That tetramer+ cells do not change in number in vaccinated mice with NKG7-deficient and -sufficient T cells is interesting and suggests that NKG7 affects the differentiation / cytokine production, rather than T cell expansion. As such, the panel provided to the reviewers in the point-to-point response would add value in the manuscript. As such, the question arises, whether the authors have the analogous data also for the experiment shown in Figure 2D/F and Figure 4A-C.

Response: As requested by the reviewer, we now provide the number of tetramer+ T cells for Fig. 3 in SFig. 4B in the manuscript. We also provide the number of CD4+CD44+ T cells for the CRISPR-edited T cells for Fig. 2D in SFig. 4A and the number of tetramer+ T cells for Fig. 4A-C in SFig. 4C and mention the results in the text.

Query 4: Two things I missed when reviewing the original version of the manuscript:
a) cfu data in Figure 4D (4C in the original version), which were log-transformed, should be displayed in a dot plot without bars. The point of a bar graph is to let people compare heights or areas of the bars. Since zero can't be shown for log-transformed values (or on a log scale), the begin of the y-axis is arbitrary, and such the size/volume of the bars, which can be misleading and thus should not be used.
b) The procedure how animals have been vaccinated and challenged are not described in the methods section in the supplemental material file.

Response: A) We modified the CFU graph in Fig. 4D according the reviewers suggestion. B) We now provide the vaccination and infection details in the revised Methods section.

We hope that the Editors agree that the paper is stronger and is now suitable for publication.

Sincerely yours,

Marcel Wuethrich, PhD
