# Supplementary material for: Natural Killer Cell Granule Protein (NKG7) promotes the development of vaccine-induced anti-fungal Th1 cells
Source: PLoS Pathog. 2026 Jul 31;22(7):e1014187. doi: 10.1371/journal.ppat.1014187 (PMC13446730; doi:10.1371/journal.ppat.1014187)
Supplement: Peer review history

# Peer review: Decision Letter 0

**DOI:** 10.1371/journal.ppat.1014187.r001

**Authors:** Tobias M. Hohl, Robert A. Cramer

1 Jun 2026

PPATHOGENS-D-26-00997

Natural Killer Cell Granule Protein (NKG7) promotes the development of vaccine-induced anti-fungal Th1 cells

PLOS Pathogens

Dear Dr. Wüthrich,

Thank you for submitting your manuscript to PLOS Pathogens. After careful consideration, we feel that it has merit but does not fully meet PLOS Pathogens's publication criteria as it currently stands. Therefore, we invite you to submit a revised version of the manuscript that addresses the points raised during the review process.

Please submit your revised manuscript by Jul 31 2026 11:59PM. If you will need more time than this to complete your revisions, please reply to this message or contact the journal office at plospathogens@plos.org. When you're ready to submit your revision, log on to https://www.editorialmanager.com/ppathogens/ and select the 'Submissions Needing Revision' folder to locate your manuscript file.

Please include the following items when submitting your revised manuscript:

* A letter that responds to each point raised by the editor and reviewer(s). You should upload this letter as a separate file labeled 'Response to Reviewers'. This file does not need to include responses to any formatting updates and technical items listed in the 'Journal Requirements' section below.

* A marked-up copy of your manuscript that highlights changes made to the original version. You should upload this as a separate file labeled 'Revised Manuscript with Track Changes'.

* An unmarked version of your revised paper without tracked changes. You should upload this as a separate file labeled 'Manuscript'.

If you would like to make changes to your financial disclosure, competing interests statement, or data availability statement, please make these updates within the submission form at the time of resubmission. Guidelines for resubmitting your figure files are available below the reviewer comments at the end of this letter.

As the corresponding author, your ORCID iD is verified in the submission system and will appear in the published article. PLOS supports the use of ORCID, and we encourage all coauthors to register for an ORCID iD and use it as well. Please encourage your coauthors to verify their ORCID iD within the submission system before final acceptance, as unverified ORCID iDs will not appear in the published article. Only the individual author can complete the verification step; PLOS staff cannot verify ORCID iDs on behalf of authors.

We look forward to receiving your revised manuscript.

Kind regards,

Tobias M. Hohl

Academic Editor

PLOS Pathogens

Robert Cramer

Section Editor

PLOS Pathogens

Sumita Bhaduri-McIntosh

Editor-in-Chief

PLOS Pathogens

orcid.org/0000-0003-2946-9497

Michael Malim

Editor-in-Chief

PLOS Pathogens

orcid.org/0000-0002-7699-2064

**Journal Requirements:**

If the reviewer comments include a recommendation to cite specific previously published works, please review and evaluate these publications to determine whether they are relevant and should be cited. There is no requirement to cite these works unless the editor has indicated otherwise.

1) Please upload all main figures as separate Figure files in .tif or .eps format. For more information about how to convert and format your figure files please see our guidelines:

https://journals.plos.org/plospathogens/s/figures

2) We are unable to open the following Supporting Information file: SFig. 2 to Fig. 1.ai. Please kindly revise as necessary and re-upload.

3) Please amend your detailed Financial Disclosure statement. This is published with the article. It must therefore be completed in full sentences and contain the exact wording you wish to be published.

i) State what role the funders took in the study. If the funders had no role in your study, please state: "The funders had no role in study design, data collection and analysis, decision to publish, or preparation of the manuscript."

ii) If any authors received a salary from any of your funders, please state which authors and which funders..

If you did not receive any funding for this study, please simply state: u201cThe authors received no specific funding for this work.u201d

iii)Currently Funder Name ( U01 AI124299 (BK) ) is missing in Funding information. Please indicate by return email the full and correct funding information for your study and confirm the order in which they should appear

4) PLOS now requires that authors provide the original uncropped and unadjusted images underlying all blot or gel results reported in a submission’s figures or Supporting Information files. This policy and the journal’s other requirements for blot/gel reporting and figure preparation are described in detail at https://journals.plos.org/plosone/s/figures#loc-blot-and-gel-reporting-requirements and https://journals.plos.org/plosone/s/figures#loc-preparing-figures-from-image-files.

When you submit your revised manuscript, please ensure that your figures adhere fully to these guidelines and provide the original underlying images for all blot or gel data reported in your submission. See the following link for instructions on providing the original image data: https://journals.plos.org/plosone/s/figures#loc-original-images-for-blots-and-gels.

In your cover letter, please note whether your blot/gel image data are in Supporting Information or posted at a public data repository, provide the repository URL if relevant, and provide specific details as to which raw blot/gel images, if any, are not available. Email us at customercare@plos.org if you have any questions.

**Reviewers' Comments:**

Reviewer's Responses to Questions

**Part I - Summary**

Please use this section to discuss strengths/weaknesses of study, novelty/significance, general execution and scholarship.

Reviewer #1: In this short report by Wuthrich and colleagues the investigators uncovered that natural killer cell granule protein (NKG7) influences antifungal Th1 responses elicited by vaccination. The authors employed robust technical approaches to examine the role of NKG7 in vaccine-induced protection against three different models of fungal infection. They report an important role for NKG7 in promoting Th1 responses, IFN-g expression, and shaping the conversion of plastic Th17 into polyfunctional memory cells. Using CRISPR-edited T cells and conditional CD4-Cre x NKG7fl/fl mice complementary approaches the authors present data to support the conclusion that  NKG7 operates intrinsically on T cells to shape Th1 responses. The findings presented are novel, supported by the data presented and will open new directions for further investigation.

Reviewer #2: This work conducted by Wuthrich et al. describes the role of Natural killer cell granule protein (NKG7) in regulating the development of vaccine-induced interferon-g (IFN-γ) producing Th1 cells, a usually protective immune response to fungal infections. They also describe the conversion of plastic Th17 cells into polyfunctional memory Th1 cells. While NKG7 has been described previously as a regulator of IFN-γ expression and resistance in experimental Leishmaniasis, the reported results are novel for fungal infections.

Their results and conclusions are supported by a battery of generated results including; 1) the linking of IFNg and NKG7 expression in multiple models of fungal vaccination; 2) the use of genetic tools including the CRISPR-editing of NKG7 in naïve, endogenous CD4+ T cells and; 3) the use of engineered conditional loss of NKG7 in mice. The introduced data are convening, the work is diligently and cleverly carried out, and the introduced work is impactful. I have very few editorial corrections and suggestions.

Reviewer #3: This short report by Wüthrich and colleagues explores the role of NKG7 in the regulation of Th1-mediated vaccine immunity against dimorphic fungi. The authors find that NKG7, which has been studied mainly in the context of NK and CD8 T cell cytotoxicity, modulates the effector function of CD4+ T cells. More specifically, they find that T cell intrinsic NKG7 promotes the development of IFN-g secreting effector T cells at the expense of Th17 cells. Open questions remain how NKG7 modulates cytokine production mechanistically (e.g. via mTORC1?) and whether NKG7 also affects the CD4 T cell longeivity as it has been proposed in case of CD8 T cells. While the authors may not solve those question in the frame of this short report, there are a few issues that need be addressed

**********

**Part II – Major Issues: Key Experiments Required for Acceptance**

Please use this section to detail the key new experiments or modifications of existing experiments that should be absolutely required to validate study conclusions.

Generally, there should be no more than 3 such required experiments or major modifications for a "Major Revision" recommendation. If more than 3 experiments are necessary to validate the study conclusions, then you are encouraged to recommend "Reject".

Reviewer #1: (No Response)

Reviewer #2: None

Reviewer #3: 1. The framing of the study is very generalized. Statements such as ‘the role of Th1 cells in antifungal resistance’ should be formulated more carefully as different Th subsets protect against different types of fungal infections.

2. Figure 2C-F: why going through the hazzle of generating NKG7-deficient CD4 T cells by CRISPR editing, rather than using CD4 T cells from NKG7 knockout mice?

3. Please provide more details and more accuracy in the results section and in the legends regarding the experimental design, mouse lines used, etc. To provide some examples: say CD4-CreERT2 throughout, not CD4-cre. Indicate how much time after vaccination mice were infected. For the latter, including schemes of the experimental schedule used in each experiment would be very helpful for the reader.

4. Figure 2D-F: According to the legend, and in contrast to the methods section, animals were analyzed 7 days post-infection. By this time point, all animals in the blue and green groups have succumbed from the infection (2E). How could T cells and cfus be analyzed at this timepoint then?

5. Figure 3D: add a summary plot for tetramer+ cells. What about fungal loads?

6. Likewise in 4C: why not show summary plots for cytokine+ populations (with individual symbols for individual animals), as done in Fig. 3?

7. The authors claim that NKG7 regulates T cell plasticity based on a shift in IFNg- vs IL-17-producing T cells at 6 months post-vaccination. Experiments with IL-17 fate reporter mice would be required to proof that NKG7-dependent IFNg production is indeed the result of plastic ex-Th17 cells. The authors should at least tune down their conclusions. The same applies to the mentioning of memory as long as they do not show any memory marker expression by the T cells.

8. All flow cytometry plots: indicate on what population the displayed plots have been gated. Provide the full gating strategy in the supplementary figures, including scatter and exclusion of dead cells and doublets.

9. All figure legends: include the number of samples per group, whether mean+SD or mean+SEM are shown, and whether data are from a single experiment or pooled from n independent experiments. The statistical tests used are abbreviated (e.g., Anova-test, instead of one-way ANOVA or two-way ANOVA)

10. “Tis” should be explained (e.g. in the sentence “Among16 distinct clusters of cells, NKG7 was expressed mainly in SC vaccinated mice by T cells that expressed IFN-g, e.g. NK-like Th1 cells (nTh1 cells), Tis cells, and clusters 1, 3, 4 and 5 of the Th1/Th17 cell spectrum”).

**********

**Part III – Minor Issues: Editorial and Data Presentation Modifications**

Please use this section for editorial suggestions as well as relatively minor modifications of existing data that would enhance clarity.

Reviewer #1: 1) Figure legends for figures 2-4 are lacking details on how many mice were used per group, how many times were the experiments performed. Details on what statistical analysis was applied to each panel are not fully described for all data sets presented. Please revise and add this important information.

2) Figure 2A shows a line between Vaccinated NKG7+ mice and vaccinated NKG7- mice together with an asterisk to denote statistical significance. Were the other groups compared for statistically significant differences? The naive NKG7- groups appears to have about a log less fungal burden that the WT naïve control. Please clarify in the legend.

3) The authors mentioned that targeting NKG7 did not affect frequency of tetramer+ cells after vaccination. Did they quantitate total numbers of antigen-specific cells?

4) For the studies with adoptive transfer of CRISP edited NKG7+ or NKG7- into RAG hosts, did the authors examine whether the frequency and total number of vaccine-elicited T cells was comparable regardless of NKG7 expression?

5) Do the authors have a hypothesis on how NKG7 is acting to shape IFN-g expression? Several studies on CD8 T cells and in a model of Leishmania are cited but I was left wondering about how is NKG7 mediating the effects reported in the current study. I understand that experimentation to address this is beyond the scope of the current study but it would be helpful to include some thoughts about how it could be acting.

Reviewer #2: 1. How is the gene deletion reported in the text at 90% while it is reported in the figure 2 legend that the actual loss of the gene likely exceeds 80% of the transcript?

2. Please spellout GCP-MSA. Specifically, what is MSA?

3. Are the data presented in Fig2A represents an infection with B. dematitidis, while Fig2B represents an infection with C. posadasii?

4. In Figure 2F, please specify what day post infection the lung CFU were processed. The same thing applies to SFig. 1A

5. There is no mention of Fig. 3B in the text.

Reviewer #3: (No Response)

**********

PLOS authors have the option to publish the peer review history of their article (what does this mean?). If published, this will include your full peer review and any attached files.

If you choose “no”, your identity will remain anonymous but your review may still be made public.

**Do you want your identity to be public for this peer review?**  For information about this choice, including consent withdrawal, please see our Privacy Policy.

Reviewer #1: No

Reviewer #2: No

Reviewer #3: No

[NOTE: If reviewer comments were submitted as an attachment file, they will be attached to this email and accessible via the submission site. Please log into your account, locate the manuscript record, and check for the action link "View Attachments". If this link does not appear, there are no attachment files.]

**Figure resubmission:**

-->While revising your submission, we strongly recommend that you use PLOS’s NAAS tool (https://ngplosjournals.pagemajik.ai/artanalysis) to test your figure files. NAAS can convert your figure files to the TIFF file type and meet basic requirements (such as print size, resolution), or provide you with a report on issues that do not meet our requirements and that NAAS cannot fix.

After uploading your figures to PLOS’s NAAS tool - https://ngplosjournals.pagemajik.ai/artanalysis, NAAS will process the files provided and display the results in the "Uploaded Files" section of the page as the processing is complete. If the uploaded figures meet our requirements (or NAAS is able to fix the files to meet our requirements), the figure will be marked as "fixed" above. If NAAS is unable to fix the files, a red "failed" label will appear above. When NAAS has confirmed that the figure files meet our requirements, please download the file via the download option, and include these NAAS processed figure files when submitting your revised manuscript.

**Reproducibility:**

To enhance the reproducibility of your results, we recommend that authors of applicable studies deposit laboratory protocols in protocols.io, where a protocol can be assigned its own identifier (DOI) such that it can be cited independently in the future. Additionally, PLOS ONE offers an option to publish peer-reviewed clinical study protocols. Read more information on sharing protocols at https://plos.org/protocols?utm_medium=editorial-email&utm_source=authorletters&utm_campaign=protocols

# Author comment: Author response to Decision Letter 1

**DOI:** 10.1371/journal.ppat.1014187.r002

8 Jun 2026

Attachment Submitted filename: 2.Rebuttal letter NKG7- final.docx

# Peer review: Decision Letter 1

**DOI:** 10.1371/journal.ppat.1014187.r003

**Authors:** Tobias M. Hohl, Robert A. Cramer

8 Jul 2026

PPATHOGENS-D-26-00997R1

Natural Killer Cell Granule Protein (NKG7) promotes the development of vaccine-induced anti-fungal Th1 cells

PLOS Pathogens

Dear Dr. Wüthrich,

Thank you for submitting your manuscript to PLOS Pathogens. After careful consideration, we feel that it has merit but does not fully meet PLOS Pathogens's publication criteria as it currently stands. Specifically, Reviewer 3 has raised minor points related to data presentation. Therefore, we invite you to submit a revised version of the manuscript that addresses the points raised during the review process.

Please submit your revised manuscript by August 1, 2026. If you will need more time than this to complete your revisions, please reply to this message or contact the journal office at plospathogens@plos.org. When you're ready to submit your revision, log on to https://www.editorialmanager.com/ppathogens/ and select the 'Submissions Needing Revision' folder to locate your manuscript file.

Please include the following items when submitting your revised manuscript:

* A letter that responds to each point raised by the editor and reviewer(s). You should upload this letter as a separate file labeled 'Response to Reviewers'. This file does not need to include responses to any formatting updates and technical items listed in the 'Journal Requirements' section below.

* A marked-up copy of your manuscript that highlights changes made to the original version. You should upload this as a separate file labeled 'Revised Manuscript with Track Changes'.

* An unmarked version of your revised paper without tracked changes. You should upload this as a separate file labeled 'Manuscript'.

If you would like to make changes to your financial disclosure, competing interests statement, or data availability statement, please make these updates within the submission form at the time of resubmission. Guidelines for resubmitting your figure files are available below the reviewer comments at the end of this letter.

As the corresponding author, your ORCID iD is verified in the submission system and will appear in the published article. PLOS supports the use of ORCID, and we encourage all coauthors to register for an ORCID iD and use it as well. Please encourage your coauthors to verify their ORCID iD within the submission system before final acceptance, as unverified ORCID iDs will not appear in the published article. Only the individual author can complete the verification step; PLOS staff cannot verify ORCID iDs on behalf of authors.

We look forward to receiving your revised manuscript.

Kind regards,

Tobias M. Hohl

Academic Editor

PLOS Pathogens

Robert Cramer

Section Editor

PLOS Pathogens

Sumita Bhaduri-McIntosh

Editor-in-Chief

PLOS Pathogens

orcid.org/0000-0003-2946-9497

Michael Malim

Editor-in-Chief

PLOS Pathogens

orcid.org/0000-0002-7699-2064

**Additional Editor Comments (if provided):**

The authors should address minor comments by Reviewer 3.

**Journal Requirements:**

If the reviewer comments include a recommendation to cite specific previously published works, please review and evaluate these publications to determine whether they are relevant and should be cited. There is no requirement to cite these works unless the editor has indicated otherwise.

1) Please amend your detailed Financial Disclosure statement. This is published with the article. It must therefore be completed in full sentences and contain the exact wording you wish to be published.

i) State what role the funders took in the study. If the funders had no role in your study, please state: "The funders had no role in study design, data collection and analysis, decision to publish, or preparation of the manuscript."

ii) If any authors received a salary from any of your funders, please state which authors and which funders..

If you did not receive any funding for this study, please simply state: u201cThe authors received no specific funding for this work.u201d

**Reviewers' Comments:**

Reviewer's Responses to Questions

**Part I - Summary**

Please use this section to discuss strengths/weaknesses of study, novelty/significance, general execution and scholarship.

Reviewer #1: The authors have properly addressed my previous questions

Reviewer #2: The authors addressed my concerns. I have no further questions and I recommend acceptance.

Reviewer #3: The authors have addressed all my concerns and carefully replied to my comments. I have a few remaining remarks

**********

**Part II – Major Issues: Key Experiments Required for Acceptance**

Please use this section to detail the key new experiments or modifications of existing experiments that should be absolutely required to validate study conclusions.

Generally, there should be no more than 3 such required experiments or major modifications for a "Major Revision" recommendation. If more than 3 experiments are necessary to validate the study conclusions, then you are encouraged to recommend "Reject".

Reviewer #1: (No Response)

Reviewer #2: See above,

Reviewer #3: (No Response)

**********

**Part III – Minor Issues: Editorial and Data Presentation Modifications**

Please use this section for editorial suggestions as well as relatively minor modifications of existing data that would enhance clarity.

Reviewer #1: (No Response)

Reviewer #2: (No Response)

Reviewer #3: Rgd my original comment #3: In Fig. 3 and Fig. 4, panels are still labelled with CD4 Cre, instead of CD4-CreERT2

Rgd my original comment #4: I understand that the authors used a separate cohort of mice for the survival experiment. But the question remains: if the data shown in Figure 2E are representative and reproducible, mice in the non-vaccinated group in and in the vaccinated -NKG7 group all succumb within 6 days. How could the analyses shown in Figure 2D and 2F be conducted on day 7, i.e. a time point when animals from two out of three groups were no longer alive.

Please also adjust the methods section regarding the time point of analysis (it currently states “Lung cells were harvested at day 4 post-infection”, which seems not to be correct ) and describe how the endpoint was defined in survival experiments (e.g. spontaneous death or culling of the animals when reaching a certain loss of weight)

Rgd my original comment #5: That tetramer+ cells do not change in number in vaccinated mice with NKG7-deficient and -sufficient T cells is interesting and suggests that NKG7 affects the differentiation / cytokine production, rather than T cell expansion. As such, the panel provided to the reviewers in the point-to-point response would add value in the manuscript. As such, the question arises, whether the authors have the analogous data also for the experiment shown in Figure 2D/F and Figure 4A-C.

Two things I missed when reviewing the original version of the manuscript:

a) cfu data in Figure 4D (4C in the original version), which were log-transformed, should be displayed in a dot plot without bars. The point of a bar graph is to let people compare heights or areas of the bars. Since zero can't be shown for log-transformed values (or on a log scale), the begin of the y-axis is arbitrary, and such the size/volume of the bars, which can be misleading and thus should not be used.

b) The procedure how animals have been vaccinated and challenged are not described in the methods section in the supplemental material file.

**********

PLOS authors have the option to publish the peer review history of their article (what does this mean?). If published, this will include your full peer review and any attached files.

If you choose “no”, your identity will remain anonymous but your review may still be made public.

**Do you want your identity to be public for this peer review?**  For information about this choice, including consent withdrawal, please see our Privacy Policy.

Reviewer #1: **Yes:**  Amariliz Rivera

Reviewer #2: No

Reviewer #3: No

[NOTE: If reviewer comments were submitted as an attachment file, they will be attached to this email and accessible via the submission site. Please log into your account, locate the manuscript record, and check for the action link "View Attachments". If this link does not appear, there are no attachment files.]

**Figure resubmission:**

-->While revising your submission, we strongly recommend that you use PLOS’s NAAS tool (https://ngplosjournals.pagemajik.ai/artanalysis) to test your figure files. NAAS can convert your figure files to the TIFF file type and meet basic requirements (such as print size, resolution), or provide you with a report on issues that do not meet our requirements and that NAAS cannot fix.

After uploading your figures to PLOS’s NAAS tool - https://ngplosjournals.pagemajik.ai/artanalysis, NAAS will process the files provided and display the results in the "Uploaded Files" section of the page as the processing is complete. If the uploaded figures meet our requirements (or NAAS is able to fix the files to meet our requirements), the figure will be marked as "fixed" above. If NAAS is unable to fix the files, a red "failed" label will appear above. When NAAS has confirmed that the figure files meet our requirements, please download the file via the download option, and include these NAAS processed figure files when submitting your revised manuscript.

**Reproducibility:**

To enhance the reproducibility of your results, we recommend that authors of applicable studies deposit laboratory protocols in protocols.io, where a protocol can be assigned its own identifier (DOI) such that it can be cited independently in the future. Additionally, PLOS ONE offers an option to publish peer-reviewed clinical study protocols. Read more information on sharing protocols at https://plos.org/protocols?utm_medium=editorial-email&utm_source=authorletters&utm_campaign=protocols

# Author comment: Author response to Decision Letter 2

**DOI:** 10.1371/journal.ppat.1014187.r004

15 Jul 2026

Attachment Submitted filename: Rebuttal letter July 14 final.docx

# Editor report: Decision Letter 2

**DOI:** 10.1371/journal.ppat.1014187.r005

**Authors:** Tobias M. Hohl, Robert A. Cramer

17 Jul 2026

Dear Dr. Wüthrich,

We are pleased to inform you that your manuscript 'Natural Killer Cell Granule Protein (NKG7) promotes the development of vaccine-induced anti-fungal Th1 cells' has been provisionally accepted for publication in PLOS Pathogens.

Before your manuscript can be formally accepted you will need to complete some formatting changes, which you will receive in a follow up email. A member of our team will be in touch with a set of requests.

Please note that your manuscript will not be scheduled for publication until you have made the required changes, so a swift response is appreciated.

IMPORTANT: The editorial review process is now complete. PLOS will only permit corrections to spelling, formatting or significant scientific errors from this point onwards. Requests for major changes, or any which affect the scientific understanding of your work, will cause delays to the publication date of your manuscript.

Should you, your institution's press office or the journal office choose to press release your paper, you will automatically be opted out of early publication. We ask that you notify us now if you or your institution is planning to press release the article. All press must be co-ordinated with PLOS.

Thank you again for supporting Open Access publishing; we are looking forward to publishing your work in PLOS Pathogens.

Best regards,

Tobias M. Hohl

Academic Editor

PLOS Pathogens

Robert Cramer

Section Editor

PLOS Pathogens

Sumita Bhaduri-McIntosh

Editor-in-Chief

PLOS Pathogens

orcid.org/0000-0003-2946-9497

Michael Malim

Editor-in-Chief

PLOS Pathogens

orcid.org/0000-0002-7699-2064

***********************************************************

The authors have addressed the remaining points by Rev. 3. The manuscript is suitable for publication

Reviewer Comments (if any, and for reference):

# Editor report: Acceptance letter

**DOI:** 10.1371/journal.ppat.1014187.r006

**Authors:** Tobias M. Hohl, Robert A. Cramer

Dear Dr. Wüthrich,

We are delighted to inform you that your manuscript, "Natural Killer Cell Granule Protein (NKG7) promotes the development of vaccine-induced anti-fungal Th1 cells," has been formally accepted for publication in PLOS Pathogens.

We have now passed your article onto the PLOS Production Department who will complete the rest of the pre-publication process. All authors will receive a confirmation email upon publication.

The corresponding author will soon be receiving a typeset proof for review, to ensure errors have not been introduced during production. Please review the PDF proof of your manuscript carefully, as this is the last chance to correct any scientific or type-setting errors. Please note that major changes, or those which affect the scientific understanding of the work, will likely cause delays to the publication date of your manuscript. Note: Proofs for Front Matter articles (Pearls, Reviews, Opinions, etc...) are generated on a different schedule and may not be made available as quickly.

Soon after your final files are uploaded, the early version of your manuscript, if you opted to have an early version of your article, will be published online. The date of the early version will be your article's publication date. The final article will be published to the same URL, and all versions of the paper will be accessible to readers.

For Research Articles, you will receive an invoice from PLOS for your publication fee after your manuscript has reached the completed accept phase. If you receive an email requesting payment before acceptance or for any other service, this may be a phishing scheme. Learn how to identify phishing emails and protect your accounts at https://explore.plos.org/phishing.

Thank you again for supporting open-access publishing; we are looking forward to publishing your work in PLOS Pathogens.

Best regards,

Sumita Bhaduri-McIntosh

Editor-in-Chief

PLOS Pathogens

orcid.org/0000-0003-2946-9497

Michael Malim

Editor-in-Chief

PLOS Pathogens

orcid.org/0000-0002-7699-2064
